# The impact of parental psychological control on externalizing problem behaviors in adolescents: the sequential mediating role of basic psychological need fulfillment and sense of defeat

**DOI:** 10.3389/fpsyg.2025.1533715

**Published:** 2025-06-05

**Authors:** Yan Liu, Yueru Tang

**Affiliations:** College of Teacher Education, Urumqi Vocational University, Ürümqi, Xinjiang, China

**Keywords:** externalizing problem behaviors, parental psychological control, basic psychological need fulfillment, sense of defeat, adolescents

## Abstract

**Introduction:**

Adolescent externalizing problem behaviors (EPBs) represent a significant public health issue, signaling potential future destructive behaviors, violence, drug abuse, and criminal activity among youth. The adolescent period is notably susceptible to these behaviors. Therefore, it is crucial to analyze the multiple factors influencing EPBs and their interactive mechanisms to enable timely interventions and effective prevention strategies. This study is based on Self-Determination Theory and Defeat-Aggression Theory, hypothesizing that the fulfillment of basic psychological needs (BPNs) and the sense of defeat sequentially mediate the relationship between parental psychological control (PPC) and EPBs.

**Methods:**

Employing a class-cluster sampling method, this study selected 742 adolescents from a city in the Xinjiang Uighur Autonomous Region as participants. Data processing and analysis were conducted using SPSS 27.0. The analytical procedure commenced with descriptive statistics, including means and standard deviations, followed by Pearson correlation analysis and sequential mediation analysis. This study examines the relationship between PPC and EPBs and tests the sequential mediating roles of BPN fulfillment and sense of defeat.

**Result:**

After controlling for demographic variables, the findings are as follows: (1) PPC directly predicts EPBs; (2) PPC indirectly predicts EPBs through BPN fulfillment; (3) PPC indirectly influences EPBs by affecting the sense of defeat; (4) The sequential mediating roles of BPN fulfillment and sense of defeat also contribute to predicting EPBs from PPC, providing new insights for the prevention and intervention of these behaviors.

**Discussion:**

The results of this study demonstrate that adolescents’ EPBs are significantly associated with PPC, the fulfillment of BPNs, and a sense of defeat. To mitigate the level of EPBs among adolescents, it is crucial for parents to exercise caution in applying psychological control. Parents should create an environment that promotes autonomy, offers robust support and encouragement, and closely monitors the fulfillment of adolescents’ BPNs and their emotional well-being.

## 1 Introduction

Externalizing problem behaviors (EPBs), which represent one of the most prevalent and enduring forms of maladjustment among children and adolescents, have attracted substantial attention within the fields of developmental psychology and psychopathology (Chang et al., 2010). Unlike childhood, adolescence constitutes a critical transitional period from childhood to adulthood, characterized by substantial physiological and psychological changes. These changes make adolescence a high-risk phase for various mental and behavioral challenges, including addiction, depression, and aggression (Mao et al., 2021). Consequently, understanding the mechanisms underlying EPBs during adolescence is crucial. EPBs, also known as behavioral issues or antisocial behaviors, are maladaptive actions directed toward the external environment. These behaviors typically arise when individuals fail to adapt to challenges, leading to psychological distress and the adoption of negative coping strategies, such as aggression, rule-breaking, and addictive behaviors (Jin et al., 2012; Li et al., 2018). The family microsystem, a foundational unit within the ecological model of human development, plays a critical role in shaping children’s behaviors. Parental attitudes, practices, and interactions during child-rearing are often the root causes of such maladaptive behaviors. Although previous research has examined the links between insecure parent–child attachment and EPBs through factors such as self-esteem, personality, and individual traits, the motivational and emotional pathways through which parental psychological control (PPC) affects adolescents’ EPBs remain poorly understood. This study is at the forefront of integrating Self-Determination Theory and Defeat-Aggression Theory, proposing a novel “psychological need defeat → defeat accumulation” chain mediation pathway. This chain mediation effect suggests that family interventions targeting the need defeat stage (prior to defeat escalation) may yield superior outcomes. For example, early adolescence could benefit from parenting training programs, such as Riesch et al.’s (1993) parent–child communication program. Upstream interventions aim to modify PPC through family therapy, exemplified by Barber et al.’s (2005) autonomy-supportive training. Downstream interventions focus on regulating adolescent frustration through mindfulness-based interventions, such as Wang and Zakaria (2024) emotion regulation protocol. These interventions contrast with prior single-mediation studies that focused solely on psychological needs. By elucidating the temporal sequence between these theoretical mechanisms, our findings offer novel insights for intervention strategies aimed at reducing EPBs and promoting healthy adolescent development.

### 1.1 The impact of PPC on EPBs in adolescents

The Frustration-Aggression Hypothesis posits that frustration invariably precedes and induces aggressive behaviors in individuals. This theory fundamentally hinges on the notion of “drive,” suggesting that aggression is not an inherent trait but rather emerges from internal drive alterations incited by external frustrations. In contrast, Self-Determination Theory underscores the dynamic interplay between individuals and their social environments in shaping psychological. Within this framework, microenvironments, especially familial contexts, are crucial for psychological maturation. A specific form of environmental control, known as psychological control, typically evokes negative emotions and maladaptive behaviors, as it compels individuals to act under coercion rather than autonomy. This type of control is characterized by intrusive parenting practices that manipulate the emotional lives of children through strategies such as emotional neglect, love withdrawal, and guilt induction, thereby stifling emotional expression and demanding compliance. It manifests in three distinct dimensions: authoritarian intrusion, love withdrawal, and psychological entanglement (Barber, 1996). In cultural contexts, particularly within Chinese families, there is a pronounced prevalence of psychological control, which can be attributed to deeply ingrained values of filial piety and Confucian philosophy that historically sanction such practices. Despite this, there has been a notable shift in recent years as scholars and society increasingly scrutinize this covert yet detrimental parenting style, prompted by a growing focus on family education (Chen et al., 2023). Empirical studies robustly associate PPC with behavioral maladj.stment. For instance, verbal hostility, such as scolding, has been shown to reinforce aggressive behavioral patterns. Additionally, psychological control contributes to a sense of insecurity, subsequently escalating tendencies toward bullying, and directly increases the likelihood of adolescent behavioral problems. Longitudinal research also demonstrates that both maternal and paternal psychological control are predictive of internalizing and externalizing problems during middle childhood (Wu C. et al., 2022; Wu W. et al., 2022). Furthermore, stable family structures have been identified as mitigating factors against EPBs. Moreover, psychological control has been found to indirectly influence EPBs through impaired volitional control (Wang et al., 2024). While existing research emphasizes the critical role of PPC in fostering EPBs, the motivational and emotional mechanisms that mediate these relationships remain inadequately elucidated. This study seeks to integrate Self-Determination Theory with the Frustration-Aggression Hypothesis to explore the underlying mechanisms of this relationship, specifically examining the mediating roles of basic psychological need (BPN) frustration and accumulated frustration.

### 1.2 The mediating role of BPN fulfillment

The theory of BPNs posits that these needs represent inherent and intrinsic psychological motivators that encompass the dimensions of competence, relatedness, and autonomy. These needs are deemed essential “nutrients” for individual growth and development. Although these psychological needs are inherent and intrinsic, the degree to which they are satisfied is significantly influenced by the individual’s environmental context and is closely linked to their psychological well-being. When parents exert excessive psychological control, it often creates an environment characterized by criticism, hostility, and control. This environment can lead to a reduction in the child’s autonomy, diminished feelings of competence, and a thwarting of their needs for close relationships, thereby adversely affecting their normal growth and development (Wang et al., 2007). In extreme cases, it may culminate in behaviors that contravene moral norms or disrupt social conventions. Furthermore, negative family dynamics, such as emotional abuse, are significantly negatively correlated with levels of BPN fulfillment (Bu et al., 2017). Conversely, parental autonomy support is positively correlated with adolescents’ levels of BPN fulfillment (Peng X. et al., 2021; Peng S. et al., 2021). When parental control behaviors, particularly negative ones such as psychological control, fail to meet children’s psychological needs, they are likely to lead to maladaptive outcomes (Deng et al., 2001). Negative parent–child relationships that frustrate BPN fulfillment may thus trigger problematic behaviors. Therefore, this study hypothesizes that BPN fulfillment mediates the relationship between PPC and EPBs in adolescents.

### 1.3 The mediating role of sense of defeat

The sense of defeat refers to feelings of defeat and powerlessness that emerge when one’s social status, rank, and identity are profoundly undermined or lost. It encompasses two dimensions: a diminished sense of achievement and feelings of defeat. It is a sensation of loss caused by setbacks and embodies a collective feeling of disappointment, helplessness, and frustration, accompanied by feelings of resentment, loss, and depression (Gilbert and Allan, 1998). Although frustration is often experienced as a setback, the concept of defeat is more pronounced in the sense of defeat (Zhu et al., 2021). The defeat-aggression hypothesis suggests that individuals may exhibit aggressive behaviors as a result of experiencing setbacks. This hypothesis provides a contextual framework for understanding adolescents’ EPBs, suggesting that positive environmental guidance can effectively reduce the likelihood of these behaviors. Research indicates that a negative emotional atmosphere within the family can adversely affect children, increasing the probability of behavioral problems. Parents’ negative emotional expressions can directly influence their children’s emotions, leading to experiences of sense of defeat and subsequently triggering problem behaviors (Chang et al., 2022). Recent longitudinal studies have explored the link between emotional problems in adolescents and EPBs, finding a significant positive correlation between the two, aligning with previous research (Zhang and Belsky, 2022). Therefore, this study hypothesizes that the sense of defeat mediates the impact of PPC on adolescents’ EPBs.

### 1.4 The intermediary role of BPN fulfillment and sense of defeat in self-determination theory

**Figure 1:**
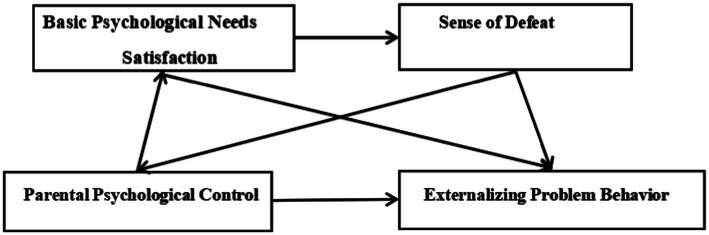
Chain intermediary model hypothesis diagram.

Self-Determination Theory asserts that self-development is an intrinsic need of every individual, foundational to the BPNs of humans. When these psychological needs are met, they promote the development of intrinsic motivation; conversely, when these needs are unmet, intrinsic motivation suffers. Self-Determination Theory differentiates between self-determined behaviors, which arise from the recognition and fulfillment of one’s needs and are characterized by autonomous action selection, and non-self-determined behaviors, which are influenced by external stimuli and lack motivational quality. A specific form of non-self-determined behavior is seen in environments characterized by psychological control. Excessive parental control can lead to stress and anxiety in adolescents (Yu et al., 2023), while parental marital conflicts are associated with lower fulfillment of BPNs in middle school students, consequently leading to aggressive behaviors (Zhou et al., 2025). Qiu (2024) posits that psychologically controlling parenting restricts children’s autonomy, competence, and relatedness, potentially leading to emotional disorders and problematic behaviors. Luo et al. have found that psychological control by parents correlates with unmet BPNs in children, leading to diminished senses of achievement and self-worth, and increased feelings of ineffectiveness. These feelings of ineffectiveness, low achievement, and diminished self-worth are pivotal components of a sense of defeat. Thus, this study proposes a sequential mediation model (Figure 1), grounded in Self-Determination Theory and the defeat-aggression hypothesis, to explore the mediating roles of BPN fulfillment and sense of defeat in the relationship between PPC and adolescents’ EPBs.

## 2 Research methods

### 2.1 Participants and procedure

This study utilized a questionnaire survey method, involving 805 students from grades 7 to 9 in City S. To ensure data accuracy and validity, questionnaires exhibiting missing values, extreme consistency, or patterned responses were discarded. This process resulted in 742 valid questionnaires, achieving an effectiveness rate of 92.17%. The participant ages ranged from 12 to 16 years, with a mean age of 14.2 years. The questionnaires were completed anonymously, and were collected on-site by the principal investigator. The demographic distribution of the participants is detailed in Table 1.

**Table 1:** Demographic information of participants (*N* = 742).

Demographic variables	Category	Count	Percentage (%)
Gender	Male	381	51.40%
	Female	360	48.60%
Grade	Grade 1	316	42.60%
	Grade 2	284	38.30%
	Grade 3	142	19.10%
Father’s educational level	Elementary school or below	149	20.10%
	Junior high school	348	47.10%
	Technical secondary school or high school	166	22.30%
	College degree or above	78	10.50%
Mother’s educational level	Elementary school or below	158	21.20%
	Junior high school	308	41.50%
	Technical secondary school or high school	192	25.80%
	College degree or above	84	11.30%
Father’s occupation	With a stable job	579	76.80%
	Without a stable job	172	23.10%
Mother’s occupation	With a stable job	579	76.80%
	Without a stable job	172	23.10%

### 2.2 Research instruments

#### 2.2.1 PPC questionnaire (parents control scale)

The PPC subscale of the Chinese version of the Parental Control Questionnaire, as developed by Wang et al., was employed to assess PPC. This scale is composed of 18 items, which are rated on a 5-point Likert scale ranging from 1 (“strongly disagree”) to 5 (“strongly agree”), where higher scores denote greater perceived PPC. It evaluates three primary dimensions: Guilt Induction (10 items, for example, “When I fail to meet my parents’ expectations, they express their disappointment”), Love Withdrawal (5 items, for example, “If I act contrary to my parents’ preferences, they become distant and aloof”), and Authoritarian Intrusion (3 items, for example, “My parents assert that their decisions are optimal for me and that I should not challenge them”). The Cronbach’s *α* reliability coefficient for this scale in the current study was 0.94.

#### 2.2.2 Chinese version of the basic psychological needs scale (C-BPNS)

The revised Chinese version of the C-BPNS, as validated by Liu et al. (2013), was utilized to gauge BPNs among adolescents. This 19-item scale encompasses three dimensions: Autonomy Need (for instance, “In daily life, I frequently feel free to express myself”), Competence Need (for instance, “Recently, I felt adept at acquiring new skills”), and Relatedness Need (for instance, “The people around me are approachable and amicable”). Responses were elicited on a 7-point Likert scale from 1 (“strongly disagree”) to 7 (“strongly agree”). The Cronbach’s *α* reliability coefficient in this study was 0.82.

#### 2.2.3 The defeat scale

The Defeat Scale, initially developed by Gilbert et al. and later revised by Tang et al. (2019), was administered to measure feelings of defeat and low achievement. This 16-item scale includes two dimensions: Low Sense of Achievement (for example, “Overall, I consider myself a winner” [reverse-scored]) and Defeat (for example, “I have been unsuccessful in achieving significant life goals”). The scale utilizes a 5-point Likert scale, ranging from 0 (“never”) to 4 (“always”), and includes three reverse-scored items (Items 2, 4, and 9). The total score can range from 0 to 64, with higher scores reflecting a stronger sense of psychological defeat. The Cronbach’s *α* reliability coefficient in this study was 0.92.

#### 2.2.4 Adolescent EPBs questionnaire

The Adolescent EPBs Questionnaire, formulated by Zhang et al. (2009) within the context of Chinese culture, was applied to evaluate EPB problems. This 14-item scale assesses three domains: Aggression (for instance, “When upset, I tend to hit walls or other objects”), Rule-breaking (for instance, “I perceive most school rules as unjust”), and Addiction (for instance, “Smoking serves merely as a distraction from ennui”). The scale is rated using a 5-point Likert scale from 1 (“strongly disagree”) to 5 (“strongly agree”), with higher scores indicating more severe EPBs. The Cronbach’s *α* reliability coefficient in this study was 0.89.

## 3 Statistical methods

### 3.1 Common method bias

Data for this study were collected through self-reported measures from participants, potentially subject to their personal response styles and traits. To evaluate this bias, Harman’s single-factor test was utilized. The analysis identified 12 principal components; notably, the first component accounted for only 23.88% of the total variance, which falls below the critical threshold of 40%. This result suggests that common method bias is not a significant concern in this study.

### 3.2 Descriptive statistical analysis of each variable

An exhaustive descriptive statistical analysis was conducted on four key variables and their sub-dimensions. As detailed in Table 2, it appears that parents tend to adopt power-assertive strategies more frequently in exerting psychological control. Regarding the fulfillment of children’s BPNs, there is a pronounced emphasis on competence needs over relational needs. With respect to experiences of frustration, children’s scores on low achievement are noticeably higher than those on feelings of defeat. Concerning externalized behaviors, the propensity for rule-breaking is most prevalent, followed by aggression, with addiction being the least common.

**Table 2:** Descriptive statistical results of each variable (*N* = 742).

Variables	*M*	*SD*
Authoritarian intrusion	2.85	1.49
Love withdrawal	2.19	1.46
Guilt induction	2.15	1.34
PPC	2.42	0.95
Autonomy need	4.41	1.92
Competence need	4.58	1.85
Relatedness need	4.30	2.09
BPNs	4.44	0.96
Low sense of achievement	2.09	1.26
Defeat	0.95	1.13
Sense of defeat	1.17	1.16
Rule-breaking	1.53	0.94
Addiction	1.09	0.47
Aggression	1.42	0.92
EPBs	1.37	0.81

### 3.3 Analysis of correlation between variables

This investigation employed Pearson correlation analysis to thoroughly examine the relationships among PPC, fulfillment of BPNs, experiences of frustration, and EPBs, as presented in Table 3.

**Table 3:** Descriptive statistics and correlation analysis of variables.

Variables	1	2	3	4	5	6	7	8	9	10
1 Gender	—									
2 Grade	0.03	—								
3 Father’s education level	−0.01	0.01	—							
4 Mother’s education level	−0.04	−0.06	0.45**	—						
5 Father’s occupation	0.06	−0.03	−0.13	−0.07*	—					
6 Mother’s occupation	0.03	−0.05	−0.07	−0.11**	0.34**	—				
7 PPC	−0.02	0.03	−0.07*	−0.06	0.03	0.13**	—			
8 BPNs	−0.08*	−0.01	0.15**	0.10**	−0.05	−0.12**	−0.31**	—		
9 Sense of defeat	0.16**	0.05	−0.13**	−0.06	0.10**	0.14**	0.48**	−0.51**	—	
10 EPBs	0.03	0.06	−0.12*	−0.04	0.03	0.09*	0.39**	−0.36**	0.54**	—
*M*	0.51	1.77	2.23	2.27	1.24	1.32	2.42	4.44	1.17	1.38
SD	0.50	0.75	0.89	0.92	0.45	0.47	0.95	0.96	0.75	0.51

*p < 0.05, **p < 0.01.

The results of the correlation analysis (as shown in Table 3) indicate a significant positive correlation between PPC and E*P*Bs (*r* = 0.39, *p* < 0.01). This suggests that an increase in PPC is likely to exacerbate EPBs. Concurrently, there is a significant negative correlation between the sense of defeat and the fulfillment of BPNs (*r* = −0.51, *p* < 0.01), revealing that lower fulfillment of BPNs may intensify the individual’s sense of defeat. Moreover, fulfillment of BPNs is significantly negatively correlated with both PPC and EPBs (*r* = −0.31, *p* < 0.01; *r* = −0.36, *p* < 0.01), implying that satisfying BPNs helps reduce both PPC and EPBs. Additionally, the sense of defeat shows significant positive correlations with both PPC and EPBs (*r* = 0.48, *p* < 0.01; *r* = 0.54, *p* < 0.01), further elucidating the positive link between the sense of defeat and these variables. These findings provide important evidence for a deeper understanding of the interactions among these variables.

Moreover, gender displays a significant negative correlation with the fulfillment of BPNs and a significant positive correlation with both the sense of defeat and EPBs. Paternal education level correlates positively with PPC, the sense of defeat, and EPBs, and negatively with the fulfillment of BPNs. In contrast, maternal education level is positively associated only with the fulfillment of BPNs. The fathers’ profession significantly correlates with the children’s sense of defeat, whereas the mothers’ profession shows a negative correlation with PPC, the sense of defeat, and EPBs, and a positive correlation with the fulfillment of BPNs.

### 3.4 Sequential mediation effect mediation effect of PPC on adolescents’ EPBs

Based on the results of the correlation analysis, this study further examined the chain mediation effects of basic psychological needs satisfaction and sense of defeat in the relationship between parental psychological control and externalizing problem behaviors. Variables were standardized prior to analysis. The mediation effects were tested using Model 6 in the PROCESS macro, with the significance of the mediation effects assessed via the bias-corrected non-parametric percentile Bootstrap method (5,000 resamples).”Use the Bootstrap method (5,000 samples) to calculate the mediation effect to reduce the influence of data skewness on the results.” The analysis treated externalizing problem behaviors as the dependent variable, parental psychological control as the independent variable, and basic psychological needs satisfaction and sense of defeat as mediators. A direct predictive effect test of parental psychological control on externalizing problem behaviors was also conducted.

After standardizing all variables and controlling for demographic covariates, regression analyses were performed with parental psychological control as the predictor, externalizing problem behaviors as the outcome variable, and basic psychological needs satisfaction and sense of defeat as mediators. The results showed that parental psychological control significantly and positively predicted externalizing problem behaviors in middle school students (*β* = 0.39, *p* < 0.001). When mediators were included in the model (see Table 4), parental psychological control significantly: Negatively predicted basic psychological needs satisfaction (*β* = −0.30, *p* < 0.001), Positively predicted Sense of defeat (*β* = 0.47, *p* < 0.001). Basic psychological needs satisfaction significantly: Negatively predicted defeat (*β* = −0.38, *p* < 0.001). Negatively predicted externalizing problem behaviors (*β* = −0.11, *p* < 0.01). Finally, when parental psychological control, basic psychological needs satisfaction, and sense of defeat were simultaneously included as predictors: Parental psychological control retained a significant positive predictive effect on externalizing problem behaviors (*β* = 0.16, *p* < 0.001). Basic psychological needs satisfaction significantly negatively predicted externalizing problem behaviors (*β* = −0.11, *p* < 0.01). Sense of defeat exhibited a significant positive predictive effect on externalizing problem behaviors (*β* = 0.42, *p* < 0.001).

**Table 4:** Regression analysis of model variable relationships (*N* = 742).

Variable	Equation 1 (BPN fulfillment)			Equation 2 (sense of defeat)			Equation 3 (EPBs)		
	*β*	SE	*t*	*β*	SE	*t*	*β*	SE	*t*
Gender	−0.08	0.06	−2.32*	0.13	0.05	4.79***	−0.48	0.06	−1.56
Grade	−0.01	0.07	−0.31	0.05	0.06	1.64	0.03	0.06	1.22
Father’s education level	0.09	0.04	2.41*	−0.06	0.04	−1.78	0.01	0.03	0.37
Mother’s education level	0.04	0.04	1.19	0.04	0.04	1.31	−0.01	0.03	−0.09
Father’s occupation	0.01	0.08	0.39	0.05	0.07	1.73	0.01	0.07	0.03
Mother’s occupation	−0.08	0.07	−2.05*	0.03	0.04	0.97	−0.03	0.06	−0.82
PPC	−0.30	0.03	−8.67***	0.35	0.03	11.72***	0.16	0.03	4.63***
BPN fulfillment				−0.38	0.03	−12.65***	−0.11	0.03	−3.04**
Sense of defeat							0.42	0.03	10.69***
	*R*^2^ = 0.07	*F* = 15.75***		*R*^2^ = 0.40	*F* = 62.82***		*R*^2^ = 0.33	*F* = 40.05***	

*p < 0.05, **p < 0.01, ***p < 0.001.

### 3.5 Mediation effect test and construction of a sequential mediation model

The study revealed significant pairwise correlations among PPC, BPN fulfillment, sense of defeat, and EPBs. Hierarchical regression analysis showed that the regression coefficients (*β*) for all four variables were significant, warranting further examination of mediation effects. The specific findings are detailed in Tables 5, 6.

**Table 5:** Path coefficients.

Path	Estimate	*S.*	*P*
PPC – BPN fulfillment	−0.27	0.04	<0.001
PPC – Sense of defeat	0.33	0.03	<0.001
BPN fulfillment – Sense of defeat	−0.38	0.03	<0.001
BPN fulfillment – EPBs	−0.10	0.03	<0.01
Sense of defeat – EPBs	0.35	0.03	<0.001
PPC – EPBs	0. 12	0.03	<0.001

**Table 6:** Test of mediation effects.

Path	Effect	Boot SE	Effect ratio	Bootstrap (95%CL)	
				Lower	Upper
Ind1	0.03	0.008		0.01	0.04
Ind2	0. 12	0.018		0.08	0.15
Ind3	0.04	0.007		0.02	0.05
Indirect effect	0.18	0.022	60.0%	0.13	0.22
Direct effect	0. 12	0.032	40.0%	0.06	0.18
Total effect	0.30	0.031		0.24	0.36

Ind1 PPC → BPN fulfillment → EPBs.

Ind2 PPC → Sense of defeat → EPBs.

Ind3 PPC → BPN fulfillment → Sense of defeat → EPBs.

The analysis indicates that the direct effect of PPC on EPBs among adolescents is 0.12, with a 95% confidence interval ranging from 0.06 to 0.18. This interval does not include zero, indicating a significant direct effect. In the mediation model, BPN fulfillment and sense of defeat act as mediators between PPC and EPBs through three pathways:

First Pathway (“PPC → BPNs → EPBs”): This pathway shows an indirect effect of 0.03, with a 95% confidence interval from 0.01 to 0.04, signifying its significance.

Second Pathway (“PPC → Sense of Defeat → EPBs”): This pathway shows an indirect effect of 0.12, with a 95% confidence interval from 0.08 to 0.15, also indicating significance.

Third Pathway (“PPC → BPNs → Sense of Defeat → EPBs”): This pathway registers an indirect effect of 0.04, with a 95% confidence interval from 0.02 to 0.05, further demonstrating its significance.

**Figure 2:**
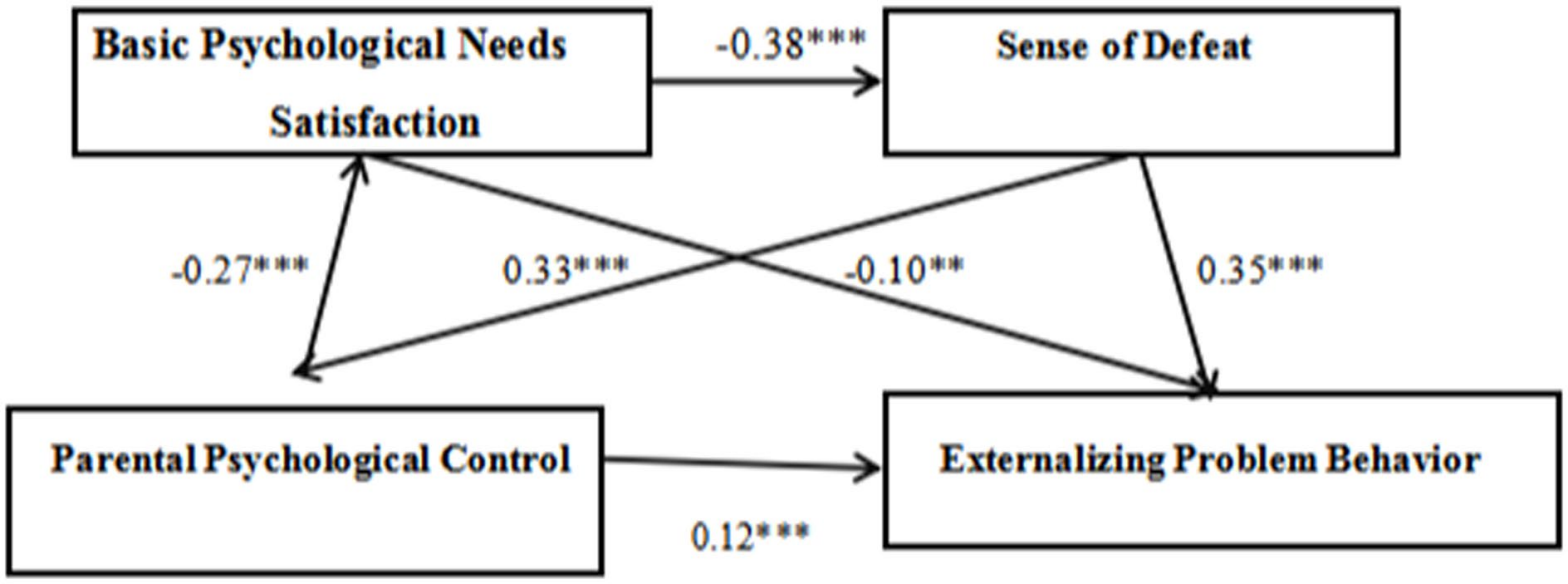
The mediating effect model of basic psychological needs satisfaction and sense of defeat on the relationship between parental psychological control on externalizing problem behaviors.

In summary, PPC can directly predict EPBs in adolescents. It also indirectly influences EPBs through BPN fulfillment and by affecting the sense of defeat. Additionally, PPC impacts EPBs through the sequential intermediary roles of BPN fulfillment and sense of defeat. The specific pathways of these intermediary effects are illustrated in Figure 2.

## 4 Discussion

This study is at the forefront of integrating Self-Determination Theory and the Defeat-Aggression Theory, proposing a “psychological need defeat → defeat accumulation” chain mediation pathway. This model elucidates the temporal sequence between mechanisms derived from these two theoretical frameworks. For instance, Kim and Chai (2019) demonstrated that PPC leads to problematic behaviors by threatening autonomy and relatedness needs, yet they overlooked emotional mechanisms. Similarly, Denson et al. (2018) identified defeat as a key proximal trigger of aggression but did not explore its distal environmental origins. Although the cross-sectional design of this study limits the exploration of dynamic causal relationships, it provides essential preliminary evidence for future longitudinal studies. For the first time, it confirms a static causal chain: environmental stimulus (psychological control) → psychological mechanism (need defeat) → emotional mechanism (defeat) → behavioral outcome (externalizing problems). This research not only advances theoretical integration but also offers nuanced guidance for selecting intervention pathways.

### 4.1 The relationship between PPC and EPBs in adolescents

Research findings demonstrate that PPC significantly predicts an increase in EPBs among middle school students, supporting our initial hypothesis. This result aligns with both domestic and international research, which shows that PPC is a strong predictor of adolescents’ EPBs (Shen et al., 2021). Furthermore, PPC is linked to higher rates of aggressive behaviors in children, including an increased likelihood of involvement in school bullying for those experiencing higher levels of psychological control. Conversely, a stable family structure has been found to mitigate these EPBs. Therefore, this study underscores the critical influence of PPC on adolescent behavioral problems, suggesting that parents should adopt evidence-based parenting strategies.

### 4.2 The mediating role of BPN fulfillment and sense of defeat in the relationship between PPC and EPBs

This study notably highlights the mediating role of BPN fulfillment in the relationship between PPC and EPBs, thereby affirming Hypothesis Two. Excessive psychological control by parents, characterized by criticism, hostility, and over-control, can impede a child’s autonomy and ability to effectively explore their environment, adversely affecting their normal developmental trajectory (Wang et al., 2007).

Additionally, the findings reveal that PPC contributes to EPBs in adolescents by inducing a sense of defeat, supporting Hypothesis Three. This aligns with the frustration-aggression hypothesis, which suggests that frustration, often a precursor to aggressive behaviors, can result from negative experiences such as defeat. Longitudinal studies further confirm a significant positive correlation between emotional problems and EPBs in adolescents, reinforcing previous findings (Zhang and Belsky, 2022; Xing et al., 2017). Consequently, PPC may intensify feelings of defeat among adolescents, heightening their risk for EPBs.

Moreover, this study confirms that PPC affects EPBs through the interconnected mediating roles of BPN fulfillment and sense of defeat, validating Hypothesis Four. Research shows a significant positive relationship between the frustration of BPNs and the sense of defeat with adverse outcomes (Huang et al., 2019). Excessive parental control and attentiveness can escalate stress and anxiety in adolescents (Yu et al., 2023), with emotional responses often tied to the fulfillment or frustration of intrinsic needs. Not all situations elicit emotional reactions; they do so when they significantly relate to an individual’s needs, leading to specific emotional responses (Lin, 2024). Depending on whether these needs are met, emotional responses can be either positive or negative. This extends the application of BPNs theory from intrinsic motivation to the mechanisms behind EPBs, suggesting that the frustration of needs (beyond mere dissatisfaction) likely leads to maladaptive behaviors through emotional pathways, complementing Deci and Ryan (2000) focus on the importance of need fulfillment for positive development.

In summary, adolescents who do not experience fulfillment of their psychological needs from their parents are more likely to encounter negative emotional states, such as feelings of defeat, thereby increasing the likelihood of engaging in problematic behaviors.

### 4.3 Educational recommendations

Based on the findings of this study, to reduce the incidence of EPBs (EPBs) among adolescents, parents should be mindful of the psychological control they exert and strive to foster autonomy by offering more support and encouragement to their children. It is also essential for parents to assess whether the BPNs of adolescents are being met and to be attentive to their emotional and affective states.

First, it is vital to provide positive and healthy family support for children’s autonomous development and social adaptation. Influenced by traditional Chinese cultural norms, an educational model that emphasizes parental authority and child compliance is prevalent in China. This model often results in parents overemphasizing authority while neglecting their children’s genuine thoughts and feelings. Parents should encourage more opportunities for independent decision-making, allowing children to take responsibility for their choices and fulfill their need for autonomy.

Second, the problem behaviors of adolescents are closely linked to the dysfunction within their family systems. Family-based CBT demonstrates significant benefits in addressing adolescents’ externalized problem behaviors, such as aggression and hyperactivity. The core mechanism of Family-CBT involves the simultaneous modification of individual behavior patterns and family interaction systems through a dual-channel intervention: behavior correction techniques and emotional regulation training. This approach addresses both individual behavior modification and the environmental factors that contribute to externalized problem behaviors at their root. This model offers a scalable ecological approach to managing adolescents’ externalized problem behaviors. By improving individual self-regulation (meeting BPNs and reducing experiences of frustration), it also enhances the protective function of the family system. Thus, mental health educators should consider not only the adolescents themselves but also the impact of their behaviors on the family system, focusing on the restoration and reconstruction of family functions to achieve more effective interventions.

Furthermore, regarding School Education Initiatives: Educators should aim to stimulate students’ intrinsic motivation for learning. Recognizing individual differences and learning conditions among students, educators should establish personalized learning demands to fully realize each student’s potential.

Moreover, concerning Emotional and Affective Development: Emotions significantly influence the learning and lives of adolescent children, as they can both enhance and impede the development of psychological and behavioral health. A collaborative effort from society, schools, and families is necessary to foster an environment that supports the enhancement of adolescents’ emotional capabilities. Concurrently, when developing emotional capability programs, schools should consider the psychological and brain development characteristics of children and adapt the curriculum to local conditions. For children facing emotional challenges, schools should also provide professional individual and group psychological support and therapy services.

### 4.4 Limitations

First, although stratified sampling was employed to account for different types of schools, unmeasured factors such as individual behavioral histories and school-specific microenvironments might still contribute to unexplained variance. Future research should utilize multilevel modeling techniques, such as HLM, to more effectively separate individual and group-level influences.

Second, while statistical analyses confirm the hypothesized pathways, the small effect sizes observed suggest that the mechanisms may have limited applicability in practical, real-world settings.

Additionally, future studies could benefit from implementing longitudinal designs, such as cross-lagged panel models, to better understand the causal and temporal relationships between variables. Expanding the sampling to include transitional areas within urban–rural gradients—such as county-level schools or schools attended by children of migrant workers—could also provide valuable insights.

Moreover, the reliance on self-reported data could introduce subjective biases. To enhance the objectivity and accuracy of measurements, future research should integrate data from multiple sources and employ methods of cross-validation.

Lastly, although our model establishes a connection between PPC, fulfillment of BPNs, frustration, and EPBs, additional mediating factors within these systems require further exploration.

## 5 Conclusion

This study investigates the influence of PPC on EPBs in adolescents within the Chinese context, highlighting the mediating roles of BPNs fulfillment and feelings of defeat. The primary findings indicate that PPC can directly predict EPBs in adolescents. It also indirectly influences EPBs through BPN fulfillment and by affecting feelings of defeat. Additionally, PPC impacts EPBs through a chain mediation involving BPN fulfillment and feelings of defeat. The unique aspects of PPC in the Chinese cultural setting are explained in light of the pressures of the Chinese educational system and the cultural norm of filial piety, particularly emphasizing the “emotional manipulation” characteristic of PPC. These results underscore the significance of the cultural context in the manifestation of adolescent behaviors and provide a foundation for future cross-cultural studies. Moreover, this research integrates Self-Determination Theory and Defeat-Aggression Theory, proposing a mediation pathway through “psychological need defeat → accumulation of defeat.” By clarifying the sequence of these theoretical mechanisms, our findings offer new perspectives for intervention strategies aimed at reducing EPBs and promoting healthy adolescent development.

## Data Availability

The original contributions presented in the study are included in the article/Supplementary material, further inquiries can be directed to the corresponding author.
