# Supplementary material for: The impact of parental psychological control on externalizing problem behaviors in adolescents: the sequential mediating role of basic psychological need fulfillment and sense of defeat
Source: Front Psychol. 2025 Jun 5;16:1533715. doi: 10.3389/fpsyg.2025.1533715 (PMC12178063; doi:10.3389/fpsyg.2025.1533715)
Supplement: Supplementary file 2 [file Data_Sheet_1.PDF]

The following is the operation process of generating descriptive statistics (including mean, standard deviation, skewness and kurtosis) and verifying data distribution in SPSS. Screenshot examples and precautions are attached:

Step 1: Generate descriptive statistics (skewness, kurtosis)

describe

[Dataset 1] E:\360MoveData\Users\Administrator\Desktop\Topic History\Total 742 data significant pure data has been reversed scored.

Descriptive statistics

	N	Minimum value statistic	Maximum value statistic	Average statistics	Standard Deviation Statistics	Polarity		Peak Temperature	
	Statistic					Statistics	Standard Errors	Statistics	Standard Error
Acts against externalization	742	14	64	19.29	7.190	2.376	.090	7.490	.179
Number of active cases (listed)	742								

Key considerations

Skewness/kurtosis threshold: Normal distribution:

absolute value of skewness <2, absolute value of peakedness <7 (ref Curran et al., 1996).

G Graph

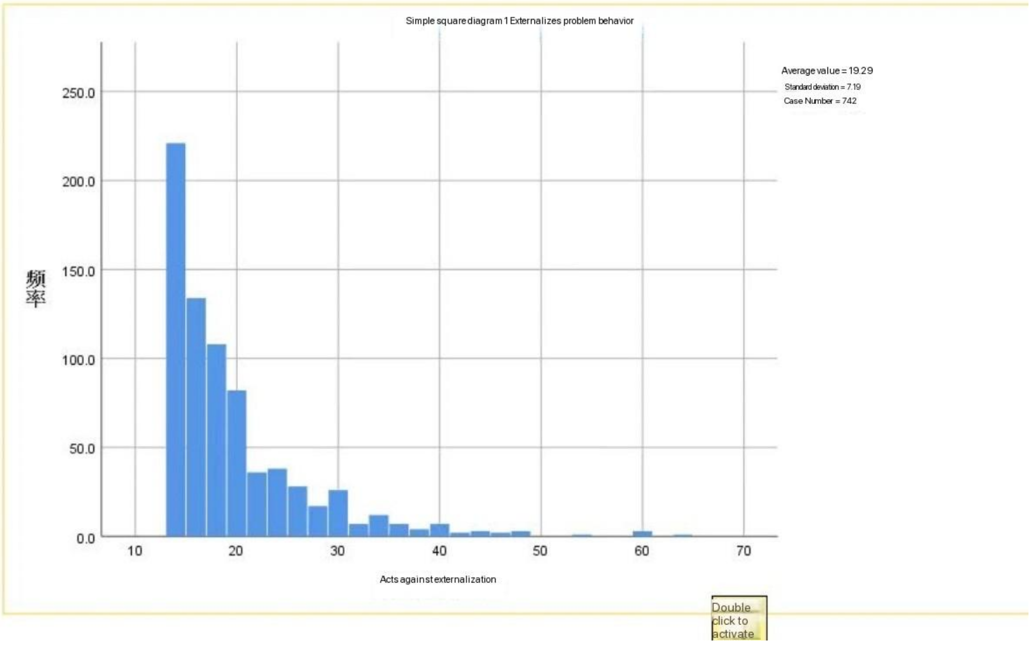

Step 2: Check EPB distribution (histogram/Q-Q plot)

1. Histogram drawing: Judgment criteria: The right side of the right skewed data histogram has a long tail.

2. Q-Q graph test for normality:

Results interpretation:If the data points are approximately distributed along the diagonal, they are close to normal; right-biased data points deviate from the diagonal to the upper right.

explore

Summary of Case Handling

	a case					
	effective		deficiency		total	
	N	Percentage	N	Percentage	N	Percentage
Acts against externalization	742	100.0%	0	0.0%	742	100.0%

describe

			Statistics	Standard Errors
Acts against externalization	average value		19.29	.264
	95% confidence interval for the average	lower limit	18.77	
		Upper limit	19.81	
	5% Average after cutting		18.33	
	median		17.00	
	variance		51.694	
	Standard deviation		7.190	
	least value		14	
	Maximum value		64	
	range		50	
	Quarterly distance		7	
	Polarity		2.376	.090
	Peak Temperature		7.490	.179

Test of normality

	Kolmogolov-Sminov (V)			Shapiro Wilk		
	Statistics	Freedom	saliency	Statistics	Freedom	saliency
Acts against externalization	.231	742	.000	.731	742	.000

A. Rieti significance correction

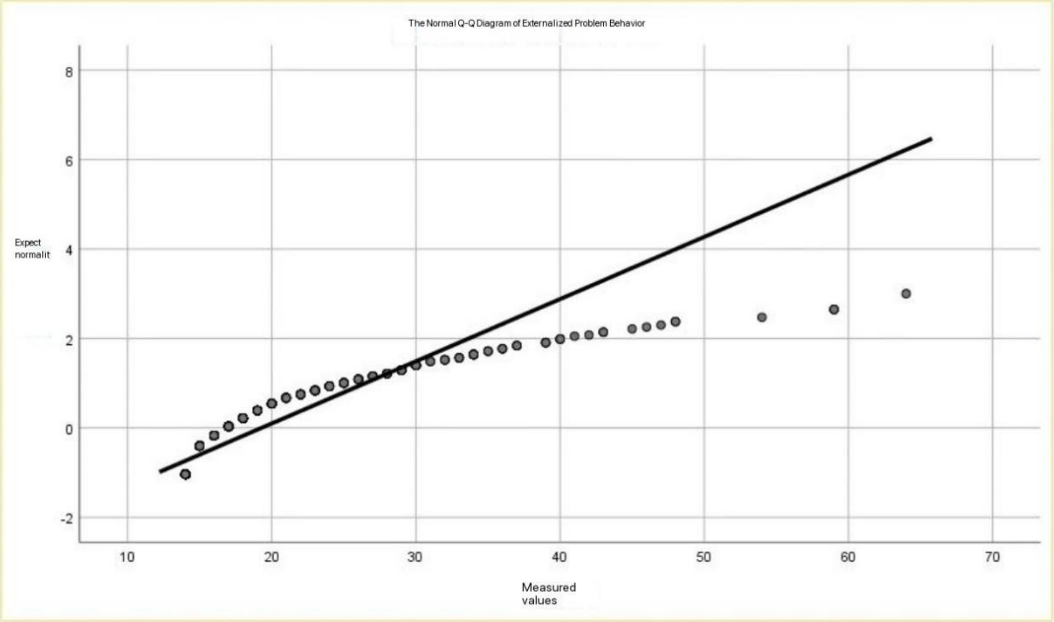

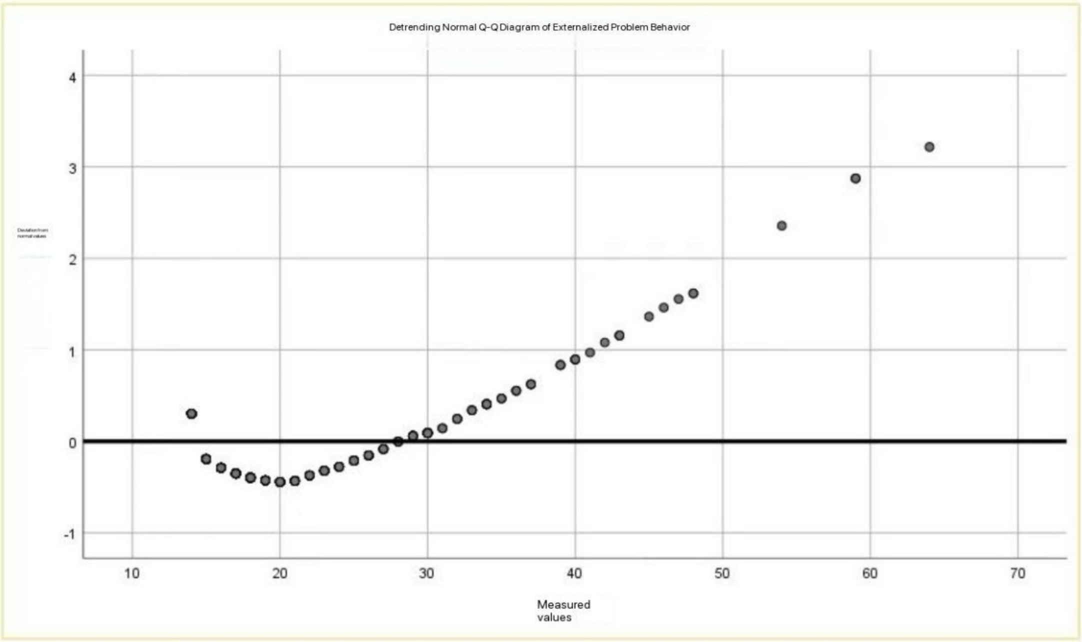

Step 3: Process skewed data (Bootstrap method)
If EPB skewness> 1, Bootstrap should be used in regression analysis:
The following is an analysis of the method used to deal with highly skewed variables (externalized problem behaviors) in PROCESS Macro Model 6:

Firstly,core methods and applicability of PROCESS model 6 Model 6 definition: Chain mediation model (two mediating variables form a chain path, that is, \ (X → M1 → M2 → Y \)). Method: PROCESS Macro based on ordinary least squares (OLS) regression.

Second, strategies and explanations for dealing with skewed distribution Strategy: Use the Bootstrap method to enhance the robustness of estimation.
operating steps:
Set `bootstrap = 5000` in the PROCESS macro (it is recommended that the sample size be greater than or equal to 5000).
Choose Bias-corrected confidence interval (more robust for asymmetric distribution).
Principle and explanation: Bootstrap Method: Construct the empirical distribution of the statistic by repeated sampling to avoid relying on the normal assumption. Applicability: Bootstrap can provide a more accurate confidence interval even if the dependent variable (EPBs) is skewed.
Results report: "Due to the highly skewed distribution of externalized problem behavior (EPBs) (skewness = 2.3, kurtosis = 7.4), we used the Bootstrap method (5000 repeated sampling) to estimate the confidence interval of the mediation effect to reduce the impact of the normality assumption."
Interpretation of the results of this paper: The output table will show the Bootstrap

confidence interval. If the interval does not contain 0, it means that the effect is significant.

Table 6 Test of Mediation Effects

Path	Effect	Boot SE	Effect Ratio	Bootstrap(95%CL)
				LOWER UPPER
Ind1	0.03	0.008		0.01 0.04
Ind2	0.12	0.018		0.08 0.15
Ind3	0.04	0.007		0.02 0.05
Indirect effect	0.18	0.022	60.0%	0.13 0.22
Direct effect	0.12	0.032	40.0%	0.06 0.18
Total effect	0.30	0.031		0.24 0.36

Note: ind1 PPC → BPN fulfillment → EPBs

ind2 PPC → Sense of defeat → EPBs

ind3 PPC → BPN fulfillment → Sense of defeat → EPBs

The data analysis indicates that the direct effect of PPC on EPBs among adolescents is 0.12, with a 95% confidence interval ranging from 0.06 to 0.18—a result that does not include zero, signifying a significant direct effect. In the mediation model, BPNs and sense of defeat serve as mediators between PPC and EPBs through three pathways:

The first pathway, "PPC → BPNs → EPBs," shows an effect of 0.03 with a 95% confidence interval from 0.01 to 0.04, indicating a significant indirect effect.

The second pathway, "PPC → Sense of Defeat → EPBs," shows an effect of 0.12 with a 95% confidence interval from 0.08 to 0.15, also indicating a significant indirect effect.

The third pathway, "PPC → BPNs → Sense of Defeat → EPBs," shows an effect of 0.04 with a 95% confidence interval from 0.02 to 0.05, further indicating a significant indirect effect.

In summary, PPC can directly predict EPBs in adolescents. It can also indirectly predict externalizing behaviors through BPNs and by influencing the sense of defeat. Moreover, it can affect EPBs through the Chain intermediary roles of BPNs and sense of defeat. Thus, BPNs and sense of defeat play chain intermediary roles in the impact of PPC on EPBs.
